# Radiotherapy induces an increase in serum antioxidant capacity reflecting tumor response

**DOI:** 10.1016/j.ctro.2024.100726

**Published:** 2024-01-11

**Authors:** F.V. Reinema, J.H.A.M. Kaanders, W.J.M. Peeters, G.J. Adema, F.C.G.J. Sweep, J. Bussink, P.N. Span

**Affiliations:** a Radiotherapy and OncoImmunology Laboratory, Department of Radiation Oncology, Radboud University Medical Center, Nijmegen, The Netherlands; b Department of Laboratory Medicine, Radboud University Medical Center, Nijmegen, The Netherlands

**Keywords:** Squamous cell carcinoma of head and neck, Reactive oxygen species, NF-E2-related factor 2 (NRF2), Antioxidants, Head and neck cancers, Oxidative stress, Oropharynx

## Abstract

**Background and purpose:**

Radiotherapy (RT) is a mainstay component of treatment for patients with head and neck squamous cell carcinoma (HNSCC), but responses vary. As RT relies upon oxidative damage, antioxidant expression in response to RT-induced reactive oxygen species (ROS) could compromise treatment response. We aimed to examine local and systemic antioxidant responses to increased RT-induced ROS in relation to treatment success.

**Materials and methods:**

Nuclear factor erythroid 2-related factor 2 (NRF2), the main antioxidant transcription factor, was immunofluorescently stained in FaDu cells and in tumor biopsies of patients with oral cavity/oropharynx HNSCC before and after five fractions of RT. Besides, total antioxidant capacity (TAC) was analyzed in HNSCC tumor cells *in vitro* and in serum of HNSCC patients before, during, and after RT.

**Results:**

Data revealed an increase in NRF2 expression and TAC in head and neck cancer cells *in vitro* over the course of 5 daily fractions of 2 Gy. In accordance, also in patients’ tumors NRF2 expression increased, which was associated with increased serum TAC during RT. Increasing serum TAC was related to impaired local tumor control.

**Conclusion:**

Radiation induced NRF2 expression and upregulated TAC, which may compromise the effect of RT-induced ROS. Changes in serum TAC during RT could serve as a novel predictor of treatment outcome in HNSCC patients.

Medical Ethics Review Committee (CMO) approval – CMO number: 2007/104.

## Highlights

• Radiotherapy increases expression of NRF2 *in vitro* and in patients’ tumors.
• Antioxidant capacity increases in serum of HNSCC patients during radiotherapy.
• Increased antioxidant capacity during radiotherapy correlates with poor local control.
• Serum antioxidant capacity could serve as a biomarker to predict treatment response.

## Introduction

As the sixth most common cancer in the world, head and neck cancers occur in the upper aerodigestive tract and are typically related to alcohol and tobacco use [1], although Human Papillomavirus (HPV) plays an important role especially in oropharyngeal cancers [2]. Of these malignancies, 90 % belong to the group of head and neck squamous cell carcinoma (HNSCC), mainly treated by surgery and radiotherapy (RT), in advanced cases combined with chemotherapy [3], [4]. RT is typically used with a treatment scheme consisting of daily fractions of 2 Gray (Gy) up to a total dose of 66–70 Gy over 5–7 weeks [1]. Although cure rates are high for early stage disease with 5-year survival rates up to 90 %, these numbers are much lower (40 %) for advanced stage HPV-negative tumors [5]. With overall about one third of patients relapsing within 2 years after treatment [4], [6], and a median survival of 9–14 months after recurrence treatment with RT [7], treatment resistance remains problematic.

RT relies upon its ionizing activity leading to the formation of free radicals such as reactive oxygen species (ROS), causing DNA double strands breaks [8]. Sensitivity to irradiation can be compromised by intrinsic tumor characteristics or be acquired during the course of treatment. Besides hypoxic tumor environments [9], p16 negativity [10], and overexpression of DNA damage repair pathways [4], acquired metabolic adaptations [11], [12], [13] can compromise tumors’ responsiveness to RT.

Recently, we reported that radioresistant cells have adapted to high levels of ROS by increasing their total antioxidant capacity (TAC) [14]. Cells displayed increased TAC which rendered them resistant to the additional ROS coming from cancer treatments such as RT. Nuclear factor erythroid 2-related factor 2 (NRF2), the main transcription factor for antioxidant gene expression [15], was increased in radioresistant breast cancer cells. Also in lung cancer cells [16], and oral squamous cell carcinoma (OSCC), NRF2 expression has been associated with radioresistance and was predictive for prognosis in advanced stage OSCC patients [17]. In HNSCC, tumoral *NFE2L2* mutations leading to stronger NRF2 activation were associated with higher locoregional recurrence after treatment with surgery and adjuvant (chemo)radiotherapy, but not after surgery alone [18].

Increased TAC within tumor cells would make tumors less responsive to ROS and inevitably render them more resistant to ROS-based therapies as RT and certain chemotherapies. Few studies have analyzed changes in TAC caused by RT. Two studies in non-tumor bearing mice report a dose dependent decrease of whole blood antioxidant capacity after whole body irradiation, while finding no changes after hind leg irradiation, suggesting no effects of local treatment on systemic TAC [19], [20]. In a cohort of cervical cancer patients treated with concurrent chemoradiation, serum antioxidant levels dropped during treatment [21]. Still, studies investigating outcome relating to serum TAC in patients treated exclusively with RT during the time of RT remain scarce.

As increased NRF2 expression was associated with a more radioresistant phenotype, we hypothesized NRF2 expression and antioxidant levels to gradually increase during the course of RT, which would compromise radiosensitivity in the long term. Our data revealed that head and neck cancer cells irradiated with 5×2 Gy developed increased NRF2 expression and TAC during the course of irradiation. Additionally, in patients, we show increased NRF2 expression in the tumor after 5 days of irradiation. For the first time we report that tumor NRF2 expression is reflected by serum TAC, which increased during the course of RT and slightly decreased after the end of treatment. This systemic increase in TAC appeared to compromise local control.

## Material and methods

### Cell culture

Human tissue derived FaDu (kindly provided by Prof Baumann, German Cancer Research Center, Heidelberg), UM-SCC-6 (kindly provided by Dr Carey, University of Pittsburgh), and UT-SCC-24A cells (kindly provided by Prof Grenman, University of Turku) were cultured in DMEM glutamax (Gibco) supplemented with 10 % fetal bovine serum, sodium pyruvate (1 mM, Gibco), nonessential amino acids (1×, Gibco), HEPES (10 mM Gibco), and penicillin/streptomycin (10 U/ml, Gibco) at 37 °C, 5 % CO_2_. Cells are regularly tested for mycoplasma. Cell lines with medium radiosensitivity (FaDu, D_37_ = 2.63 Gy; UM-SCC-6, D_37_ = 2.58; UT-SCC-24A, D_37_ = 2.36) were chosen based on previous research which also defined these lines as HPV negative [22].

### Irradiation

**Fig. 1:**
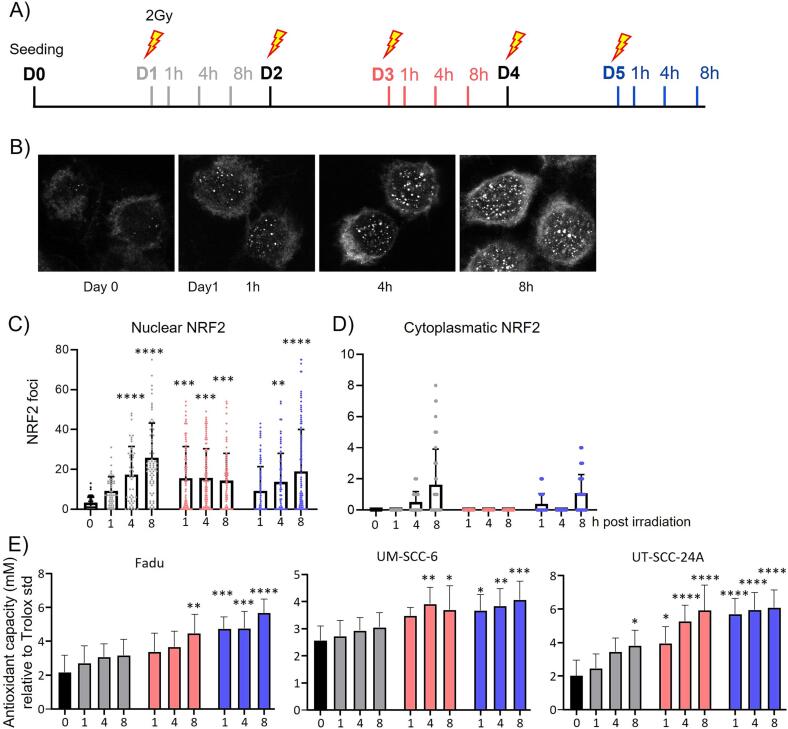
**NRF2 expression and antioxidant levels increased following irradiation *in vitro*.A)** Irradiation scheme for NRF2 staining and TAC measurement. **B)** NRF2 staining on Fadu cells. **C)** Nuclear NRF2 expression on Fadu cells. **D)** Cytoplasmatic NRF2 expression on Fadu cells. **E)** Antioxidant capacity of Fadu, UM-SCC6, UT-SCC24A cells. Data are represented as mean + SD. Color-coding of C)-E) matches A). Statistical significance was determined by Kruskal-Wallis test (C), and one-way Anova with multiple comparisons, comparing each condition to unirradiated (E). *, p < 0.05 ; **, p < 0.01; ***, p < 0.001; ****, p < 0.0001.

Cells were irradiated in T75 flasks and 2-wells chamber slides (Lab-Tek, Thermo Fisher) with 2 Gy (X-RAD Biological Irradiator; Precision X-ray) on five consecutive days (Fig. 1A). On day 1, 3, and 5, each at 1 h, 4 h, and 8 h after irradiation, cells in chamber slides were fixed for NRF2 staining, while cells in T75 flasks were prepared for antioxidant capacity analysis by sonification.

### **Clinical** study

Tumor biopsies and serum were obtained from 49 patients within a study titled: *Early treatment-induced changes in the tumor microenvironment and potential consequences for biology-based predictive tests in head and neck cancer*, approved by the Medical Ethics Review Committee (CMO) Arnhem-Nijmegen (CMO number: 2007/104). Patients provided informed consent for participation in the study. Patients treated by RT for oral cavity/oropharynx HNSCC consented to two biopsies, one before RT treatment (T0) and the second biopsy taken on day 5 of their RT course (T1; Table 1). Additionally, blood was collected on four timepoints; the two timepoints of biopsy (T0 and T1), during the last week of RT (T2) and 6–8 weeks after RT (T3). Serum was available from all four time points from 27 patients. Due to hemolytic serum from one patient, serum of 26 patients was available to measure TAC. Tumor containing biopsies from both time points were available for 14 of these patients. All patients were treated with primary RT of which seven also received concurrent Cisplatin, and two radiotherapy combined with EGFR inhibition (Cetuximab).

**Table 1:** Clinicopathological characteristics of the HNSCC patient cohort.

		n
Number of patients		27
Sex	female	8
	male	19
Average age at diagnosis		59 (32–77)
Smoking status	smoker	18
	non-smoker	6
	unknown	3
Alcohol consumption	yes	16
	no	6
	unknown	5
Primary tumor site	oropharynx	24
	oral cavity	3
T class	T2	12
	T3	6
	T4	9
N class	N0	10
	N1	4
	N2	11
	N3	2
Radiotherapy	IMRT^1^	23
	3D conformal RT^2^	4
Cisplatin		7
Cetuximab		2
Median follow-up (months)		39 (7–173)
Survival (50 months)	yes	14
	no	13
Local failure		10
Regional failure		3
Locoregional failure		12
Metastasis		2

1 68 Gy/50.3 Gy simultaneous integrated boost, 6 fractions/week, OT 5,5 weeks.

2 68 Gy/44 Gy sequential boost 5 fractions/week for 4 weeks, 10 fractions/week for 1,5 weeks, OT 5,5 weeks; OT: overall treatment time.

### NRF2 staining

*In vitro*, after irradiation, cells were fixed in paraformaldehyde (4 %), permeabilized (0.1 % Triton-X, Sigma), blocked, and stained for NRF2. Primary antibody against NRF2 (D1Z9C, Cell Signaling, dilution 1/800) was applied (45 min, room temperature). Anti-rabbit-488 (A21206, Invitrogen, dilution 1/300) was used as secondary antibody. Nuclei were counterstained with Hoechst. For each cell line, 6 images were taken randomly at a Zeiss LSM900 microscope and number of foci per nucleus, as well as number of foci per cell were quantified using Fiji/ImageJ.

Tissue sections from 14 patients were stained for NRF2 as above. Due to disturbed tissue integrity after irradiation, it was not possible to analyze foci per nucleus. Therefore, analysis was quantified as foci per image taken, with all images consisting of tumor mass only.

### Antioxidant measurement

Antioxidant capacity was measured in cell lysates and patients’ serum with the Antioxidant assay kit (CS0790, Sigma) following the manufacturer’s protocol.

### Statistical analysis

Statistical analysis was performed using GraphPad Prism 8.01 software. *In vitro* data were analyzed by one-way ANOVA with multiple comparisons after normality was confirmed, or by Kruskal-Wallis test. Patient data was analyzed by paired *t*-test, one sided *t*-test or one-way ANOVA. Kaplan-Meier survival curves were based on increased antioxidant capacity at T2 compared to T0, with a cutoff follow-up of 50 months and compared by Mantel-Cox testing. We defined TAC as increased when 10 % higher at T2 than at T0. Notably, this classification into groups was not different when choosing a cut-off of 30 %. Data is generally shown as mean +/± SD. A *p*-value < 0.05 was considered statistically significant.

## Results

### NRF2 expression and antioxidant levels increase during radiotherapy *in vitro*

First, we assessed the expression dynamics of NRF2 during the course of irradiation based on standard fractionation schedules. HNSCC FaDu cells were irradiated (5x2 Gy) and stained for NRF2 at several days and timepoints after irradiation (Fig. 1A). Images show a rapid increase of NRF2 expression after irradiation within the nuclei, which expands to the cytoplasm at several timepoints (Fig. 1B). Quantification of NRF2 foci within the nuclei showed a low baseline NRF2 expression before irradiation (3.2 foci/nucleus, Fig. 1C). Expression already increased 1 h after irradiation (9.2 foci/nucleus, p = 0.0741) and continued to increase at 4 h (17.4 foci/nucleus, p < 0.0001), and 8 h (25.8 foci/nucleus, p < 0.0001) after irradiation. RT-induced NRF2 expression was at a medium level during day 3 (around 15 foci/nucleus). After the fifth fraction, NRF2 started to increase again to an average of 19 foci/nuclei (p < 0.0001) 8 h (Fig. 1C).

NRF2 foci were not only found inside the nucleus, but also in the cytoplasm of cells at a few timepoints (4 h and 8 h at Day 1, and 8 h at day 5; Fig. 1D), following the same dynamic as the nuclear NRF2 expression.

As NRF2 is the main transcription factor of antioxidant gene expression [15], these data indicate TAC to increase following irradiation. To relate NRF2 expression to TAC, we irradiated the human HNSCC cell lines FaDu, UM-SCC-6 and UT-SCC-24A cells in the same time scheme as above (Fig. 1A) and measured TAC at these timepoints (Fig. 1E). For comparability, these three cell lines were chosen to exhibit a medium radiosensitivity as previously determined [22]. During the five fractions of 2 Gy, all three cell lines displayed gradually increasing TAC, which tripled in FaDu (from 2.1 mM to 5.6 mM; p < 0.0001) and UT-SCC-24A cells (from 2.0 mM to 6.1 mM; p < 0.0001), and almost doubled in UM-SCC-6 cells (from 2.5 mM to 4.0 mM; p = 0.0006). These data strongly suggest that tumor cells increase their antioxidant capacity in response to irradiation, indicating that protective mechanisms are activated upon irradiation.

### NRF2 is increased in tumors of head and neck cancer patients after 5 fractions of irradiation

As above *in vitro* data suggests that irradiated cancer cells can quickly activate antioxidant protective mechanisms, we aimed to examine whether the same response holds true in patients treated with RT for oral cavity/oropharynx HNSCC. Most patients received intensity modulated RT (IMRT; n = 23), with a RT scheme of 50.3/1.48 Gy and a simultaneous integrated boost to the primary tumor of 68/2 Gy, 6 fractions per week and an overall treatment time of 5,5 weeks. Two biopsies were taken, one before RT (T0), and one 5 days into treatment (T1), and blood drawn at T0, T1, during the last week of RT (T2), and 6–8 weeks post RT (T3). Biopsies were available from 14 patients, while serum was available from 26 patients, in which we analyzed NRF2 expression and TAC respectively.

**Fig. 2:**
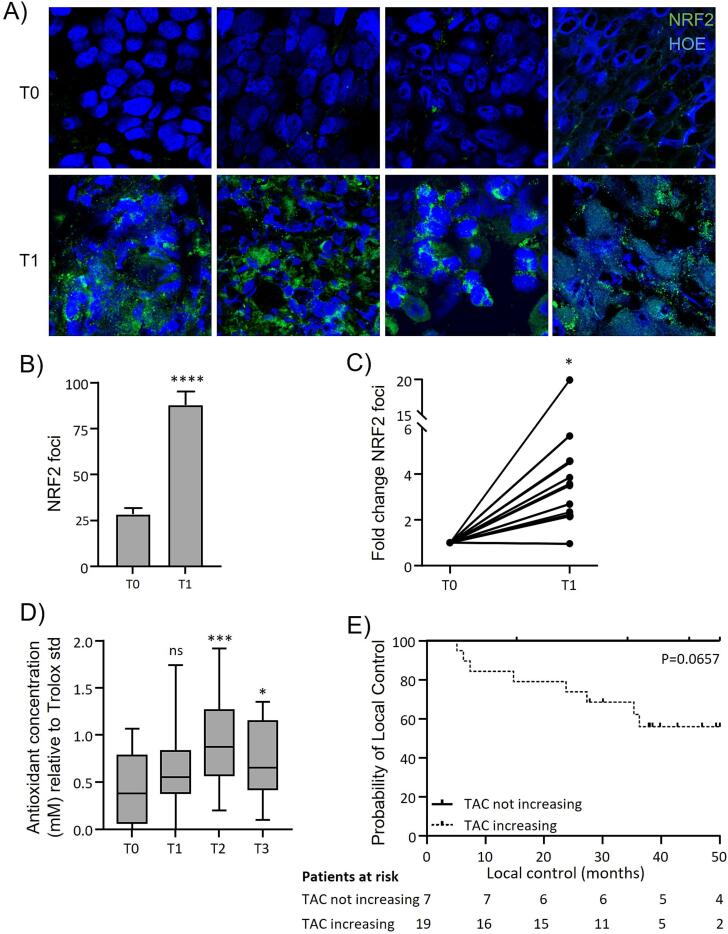
**NRF2 expression and antioxidant levels increased following irradiation in patients.A)** NRF2 staining (green) in 4 individual patients before RT (T0) and after 5 fractions (T1). **B)** Number of foci quantified from staining. **C)** Fold change NRF2 based on B) for individual patients. **D)** Antioxidants in patients' serum (n=26) before RT (T0), after 5 fractions (T1), during the last week of RT (T2) and 6-8 weeks after treatment (T3). **E)** Local disease control in patients with increased TAC at T2 and patients with steady TAC. Data are represented as mean ± SD. Statistical significance was determined by Mann-Whitney test (B), by one sample t-test (C) comparing T1 with T0, by one-way ANOVA with multiple comparison between T1, T2, and T3 testing against T0 (D), and by Mantel-Cox survival analysis (E). *, p < 0.05 ; **, p < 0.01; ***, p < 0.001; ****, p < 0.0001.

NRF2 staining (Fig. 2A) in paired biopsies showed significantly increased expression of NRF2 at T1 compared to T0 (p < 0.0001; Fig. 2B). This increase was seen in all patients in varying degrees of fold change, with the exception of one patient (p = 0.02; Fig. 2C).

### Systemic antioxidant capacity increases during the course of radiotherapy

As local RT could have systemic effects, and antioxidant capacity induced by NRF2 would attenuate treatment efficacy [14], we next investigated whether such systemic effect might be discernable in serum of 26 patients. If so, this approach might yield a biomarker that could be used to predict therapy resistance. Irradiation of head and neck tumors lead to increased antioxidant levels in the serum of patients. Antioxidant levels significantly increased up to the last week of RT (T2; p = 0.0002 compared to T0; Fig. 2D) and compared to T2 slightly decreased at T3, 6–8 weeks after RT, but remained elevated relative to T0 (p = 0.046).

We compared outcome of patients who displayed an increase in serum TAC (n = 19) to patients without increasing TAC (n = 7) at T2 compared to T0. T2 was chosen as the timepoint with the most prominent TAC increase. Analysis showed a differential probability of local control (Fig. 2E). Patients whose TAC did not increase tended to have a better local control than patients with increasing TAC. Indeed, all events were limited to the group of patients with increasing antioxidant capacity at T2, although this did not reach statistical significance due to the limited number of events leading to lack of power (p = 0.0657). Treatment success measured as local control was not influenced by the initial TAC as measured at T0 or TAC after treatment at T3.

## Discussion

RT is widely used in HNSCC, but differential treatment responses still compromise treatment success [4], [6]. As antioxidants may counteract the ROS-based effect of RT, we aimed to investigate the effect of irradiation on NRF2 expression and related antioxidant capacity *in vitro* and in patient biopsies prior to RT and during treatment. Our results demonstrate that NRF2 expression is upregulated in tumor cells *in vitro* and in patient tumors after 5x2 Gy RT. TAC was not only increased in tumor cells *in vitro*, but also systemically in patients’ serum. Survival analysis indicated that increasing serum TAC during RT is associated with impaired local control, thus radioresistance.

We used NRF2 and TAC as measured for antioxidant activation as these are the most general options to quantify antioxidant capacity. NRF2 is the main transcription factor for antioxidant expression and mediates several pathways as for instance glutathione, thioredoxin, and superoxide dismutase 1 [15]. Consecutively, TAC is a similar parameter denoting the general antioxidant response comprised by these pathways. Although these may be of interest to look at individually, here we aimed to focus on the total antioxidant response, as NRF2 would be a feasible general target for clinical inhibition to counter antioxidant-mediated ROS resistance.

Five consecutive days of irradiation *in vitro* lead to a distinct pattern of increased NRF2 expression. Nuclear expression rapidly increased in FaDu cells, indicating the ability of tumor cells to promptly respond to oxidative stress and activate protective mechanisms. This is also reflected by the subsequently increasing TAC levels in three HNSCC cell lines during 5 days of irradiation. This upregulation of NRF2 leading to higher TAC is in line with previous findings in mouse embryonic fibroblasts where antioxidants were shown to be increased after five consecutive days of 0.5, 2, or 4 Gy [23]. Interestingly, a stagnant nuclear NRF2 expression was observed on day 3, suggesting an autoregulatory mechanism. This could for instance be influenced by MnSOD, a mitochondrial antioxidant interacting with NRF2, which was shown to stabilize the antioxidant pool [24]. Besides, antioxidants could be activated by other pathways with different temporal dynamics, such as NF-κB [23], explaining these findings.

Cytoplasmic NRF2 expression might further contribute to regulating nuclear NRF2 and subsequent TAC as it has been hypothesized to reflect an important capacity to respond to stress signals [25]. In line with this, cytoplasmic NRF2 has been associated with inferior survival and poor prognosis following cisplatin chemotherapy in lung cancer patients [26]. As cisplatin is a ROS-based chemotherapy, this observation supports the hypothesis that cytoplasmic NRF2 serves as a marker for a strong response against oxidative stress, which could alter RT efficacy leading to a more resistant phenotype. Hence, the opposite effect of cytoplasmic NRF2 loss was reported to increase cellular radiosensitivity [26].

Resistance developed due to chronic RT-induced ROS exposure with subsequent NRF2-mediated upregulation of TAC would be a contributing factor towards eventual treatment failure. To investigate the described *in vitro* mechanisms in patients’ tumors during the cause of fractionated RT, we stained biopsies before and during RT for NRF2 and measured TAC in patients’ serum. Just as *in vitro*, tumoral NRF2 expression was significantly upregulated after 5 daily fractions of 2 Gy. As this expression suggests an upregulation of antioxidant expression that could negatively impact treatment response, we investigated changes in antioxidant levels in patients’ serum which could serve as a potent predictive biomarker. Indeed, serum TAC increased during the course of RT, which could display protective mechanisms against RT-dependent ROS, as hypothesized for lung cancer before [27]. Mutations in the NRF2 pathway could alter individual responses to RT and explain differences in NRF2 and TAC expression. However, only a small group of 17.4 % of head and neck cancers are associated with mutations in this pathway [28].

Serum antioxidants might originate as a systemic response from the tumor, released into the blood stream as signaling molecules [29]. On the other hand, during RT of (HNSCC) tumors, a substantial volume of blood is irradiated as well, which implies that blood cells may also respond with antioxidant production for protection. With a fraction of 1.5 Gy per irradiation (average 4 min duration), about 67 % of a patient’s blood gets irradiated, translating to a dose of 0.0375 Gy per fraction. Over a treatment course of 5.5 weeks, with 34 fractions, this would add up to 0.84 Gy. This dose is based on a simplified calculation assuming irradiation of the carotid artery and vena jugularis at a length of 15 cm, and presuming a constant dose rate and normally distributed blood flow. As this is a very low dose, directed towards the blood, effects might be negligible. Low doses of >0.1 Gy have been shown to induce activation of NRF2 and Heme oxygenase, a strong antioxidant, in mouse macrophages *in vitro* before [30]. However, whether a dose of 0.0375 Gy per fraction on blood would be enough to trigger a significant effect on serum TAC remains questionable. The body also has the capacity to store antioxidants within cells, in extracellular fluids or membranes, which can be mobilized following excess ROS. Besides, a part of antioxidants in the blood stream originate from the diet [31]. We do not have data on (antioxidant) supplements patients might have been taking, and the question of their radioprotective effects remains unsettled [32]. However, considering the fact that the increase in TAC during RT, and not TAC before RT, was indicative for poor treatment success suggests that diet had negligible effects in these results.

Independent of the origin of antioxidants, an increase of serum TAC during RT tended to compromise treatment success with a higher chance of local recurrence. Although research regarding outcome after antioxidant changes during RT is scarce, similar results were found in cervical cancer, as reduction of serum TAC during chemoradiation was shown to be beneficial for survival [21]. Local recurrence was not related to TAC before starting RT, revealing that especially the upregulation of TAC in response to RT, and therefore a protective antioxidant response compromises treatment sensitivity. As RT heavily relies on oxidative damage by ROS, this adverse effect of elevated TAC would scavenge treatment-induced ROS, protecting tissue, and compromising treatment success. Survival analysis was not significant probably due to the small cohort limiting power. Similarly, a direct correlation between NRF2 and TAC could not be established. However, a strong tendency indicates an interaction between tumor antioxidants and serum antioxidants, and an important clinical value of measuring serum TAC.

Following, increased TAC in response to RT-induced ROS could instigate treatment resistance, and compromise treatment success. In case of recurrence, cells are likely to have developed at least some resistance towards ROS-directed therapies, drastically reducing treatment options. In HNSCC, no clinical therapy-stratifying biomarkers are available. As TAC can easily and affordably be measured in patients’ serum during treatment, it could serve as a valuable marker to predict treatment outcome early into treatment and provide possibilities to adapt ongoing treatment to maximize treatment success by for instance combining therapies. Also, NRF2 inhibiting drugs are under development with the aim to (re)sensitize cells to ROS [33], which has been proven successful *in vitro* [34], [35], [36]. When incorporated in treatment adaptations, this would increase the chances of successful therapy in patients with increased TAC.

## Conclusions

We show elevated NRF2 expression and antioxidant capacity during radiotherapy *in vitro* and in patients’ tumors and serum respectively reflecting ROS-protective mechanisms being activated due to oxidative stress. Increasing serum antioxidant capacity in patients during RT showed a strong trend towards compromised local control. Therefore, it may serve as a predictor of treatment outcome during therapy.

## Declaration of competing interest

The authors declare that they have no known competing financial interests or personal relationships that could have appeared to influence the work reported in this paper.
